# Establishment and Culture of Human Intestinal Organoids Derived from Adult Stem Cells

**DOI:** 10.1002/cpim.106

**Published:** 2020-09-17

**Authors:** Cayetano Pleguezuelos‐Manzano, Jens Puschhof, Stieneke van den Brink, Veerle Geurts, Joep Beumer, Hans Clevers

**Affiliations:** ^1^ Hubrecht Institute Royal Netherlands Academy of Arts and Sciences (KNAW) and UMC Utrecht Utrecht The Netherlands; ^2^ Oncode Institute Utrecht The Netherlands; ^3^ The Princess Maxima Center for Pediatric Oncology Utrecht The Netherlands

**Keywords:** adult stem cells, human intestinal organoids, organoid cryopreservation, organoid culture establishment, organoid differentiation, organoid immunofluorescence, organoid passage, single‐cell clonal organoid culture, specialized organoid reagents

## Abstract

Human intestinal organoids derived from adult stem cells are miniature ex vivo versions of the human intestinal epithelium. Intestinal organoids are useful tools for the study of intestinal physiology as well as many disease conditions. These organoids present numerous advantages compared to immortalized cell lines, but working with them requires dedicated techniques. The protocols described in this article provide a basic guide to establishment and maintenance of human intestinal organoids derived from small intestine and colon biopsies. Additionally, this article provides an overview of several downstream applications of human intestinal organoids. © 2020 The Authors.

**Basic Protocol 1**: Establishment of human small intestine and colon organoid cultures from fresh biopsies

**Basic Protocol 2**: Mechanical splitting, passage, and expansion of human intestinal organoids

**Alternate Protocol**: Differentiation of human intestinal organoids

**Basic Protocol 3**: Cryopreservation and thawing of human intestinal organoids

**Basic Protocol 4**: Immunofluorescence staining of human intestinal organoids

**Basic Protocol 5**: Generation of single‐cell clonal intestinal organoid cultures

**Support Protocol 1**: Production of Wnt3A conditioned medium

**Support Protocol 2**: Production of Rspo1 conditioned medium

**Support Protocol 3**: Extraction of RNA from intestinal organoid cultures

## INTRODUCTION

Adult stem cell (ASC)–derived organoids are miniature versions of epithelia that grow in three dimensions and can be expanded ex vivo. These mini‐organs are derived from tissue biopsies and are entirely composed of primary epithelial cells, relying on the ability of the adult stem cells to expand indefinitely. They permit the study of epithelial physiology in a setup that closely resembles the in vivo situation. In contrast to animal models, adult stem cell organoids allow us to examine direct interactions between different cell types in a reductionist approach. Because they can be derived from humans, adult stem cell organoids capture the specific characteristics of human tissues. All these characteristics make them a powerful tool (Clevers, 2016).

Because organoids retain the characteristics of the native tissue from which they were derived, human intestinal organoids have been useful in the study of intestinal epithelial physiology and stem cell differentiation dynamics (Beumer et al., 2020 [human]; Farin et al., 2016 [mouse]). Additionally, human intestinal organoids have been used to study human disease (Fujii, Clevers, & Sato, 2019), usually following three approaches. (1) Disease modeling has been carried out by establishing organoid lines from monogenic disease patients, e.g., from cystic fibrosis (*CFTR*; Dekkers et al., 2013), multiple intestinal atresia (*TTC7A*, Bigorgne et al., 2014), congenital diarrheal disorders *(DGAT1*; van Rijn et al., 2018). By repairing these mutations using CRISPR/Cas9‐based genetic engineering in the patient‐derived organoids, this technology might be used in future regenerative medicine approaches (Geurts et al., 2020; Schwank et al., 2013). (2) Introduction of mutations, again using CRISPR/Cas9 technologies, in healthy organoids allows molecular studies of different diseases such as cancer (Drost et al., 2015; Fujii, Matano, Nanki, & Sato, 2015). (3) Establishment of tumor and matched healthy organoid lines from colorectal cancer (CRC) patients allows study of treatment response and tumor heterogeneity (Drost & Clevers, 2018; van de Wetering et al., 2015). Furthermore, organoids have proven to be valuable tools for studying mutagenic processes in human physiology and disease (Blokzijl et al., 2016; Christensen et al., 2019; Pleguezuelos‐Manzano et al., 2020).

The human intestinal epithelium functions as a physical barrier that separates the internal and external face of the intestinal tract. Because of this, the intestinal epithelium is in constant interplay with the immune environment. This interplay is highly important for maintenance of a homeostatic state, and its imbalance is often linked to disease. For this reason, human intestinal organoids have emerged as a great tool for studying the function of this epithelium and its relations with the immune component in disease (Bar‐Ephraim, Kretzschmar, & Clevers, 2019), including celiac disease (Dieterich, Neurath, & Zopf, 2020; Freire et al., 2019) and ulcerative colitis (Nanki et al., 2020) among others. Moreover, human intestinal organoids can be useful in cancer immunotherapy research (Dijkstra et al., 2018).

This article describes how to establish organoid cultures from biopsies of human small intestine or colon (Basic Protocol 1) and how expand the organoid cultures ex vivo (Basic Protocol 2). An Alternate Protocol focuses on how to differentiate expanding intestinal organoids (predominantly composed of stem cells and transit‐amplifying cells) towards a more mature cell type composition (i.e., enterocytes, enteroendocrine cells, or goblet cells). Organoid cryopreservation and thawing are discussed in Basic Protocol 3. Several methods then exemplify how to perform some of the most common downstream applications/readouts with organoids. Basic Protocol 4 describes immunofluorescence staining for fluorescence/confocal imaging. In light of the increasing importance of CRISPR/Cas9‐engineered organoids and whole‐genome sequencing (WGS) of organoid lines, a protocol is provided for single‐cell clonal outgrowth of intestinal organoids (Basic Protocol 5), which is of key importance for both applications. The production of two essential medium components, Wnt3A and Rspo1 conditioned medium, is detailed in Support Protocols 1 and 2. Finally, these protocols are supplemented by a method for extracting organoid RNA (Support Protocol 3) that can be used for gene expression readouts like quantitative real‐time PCR or RNA sequencing.

## STRATEGIC PLANNING

Organoid lines can be established from biopsies (Basic Protocol 1) or obtained from a biobank or the research community. From biopsies, organoids can normally be derived in sufficient quantities for extensive experimentation and storage within a month. Established lines can be shipped on dry ice and used for experiments 1‐2 weeks after thawing. Single‐nucleotide polymorphism fingerprinting or comparable methods should be used to confirm line identity over time. As with cell lines, mycoplasma tests should be performed regularly. The use of patient‐derived material should in all cases comply with the ethical regulations and guidelines of the relevant institutional boards. Written informed consent is required from all donors.

### General Considerations

Human organoid lines present some degree of heterogeneity due to inter‐ and intra‐donor variability. Particularly, the expansion ability or “stemness” of some lines is higher than others, and this usually inversely correlates with their ability to achieve full differentiation. This should be taken into consideration when selecting a pre‐established organoid line or when characterizing a newly established line. Furthermore, there are some differences between organoids derived from different regions of the intestinal tract, reflecting intrinsic differences between these epithelial regions in vivo. Generally, duodenal organoids show a higher stemness ex vivo compared to those from other regions, and therefore are easier to expand. A key factor to organoid culture expansion is activation of the Wnt pathway. To achieve this, two different Wnt sources (Wnt3A conditioned medium and Wnt surrogate molecules) have been successfully applied. Wnt3A conditioned medium (Wnt3A‐CM, see Support Protocol 1) is a more economical source of Wnt ligands, but yields a lower level of Wnt activation and may suffer from batch‐to‐batch variability. In contrast, synthetic Wnt surrogate (Miao et al., 2020) can achieve high activation of the Wnt pathway, fueling faster organoid expansion in some organoid systems. The use of Wnt surrogate is recommended especially for human colon organoid cultures and when growing single‐cell clonal cultures.

### Preparation and Considerations Before Starting Organoid Work

Basement membrane extract (BME) should be thawed overnight at 4°C before starting any of the protocols described. Once thawed, it should be kept at 4°C at every moment. Freeze‐thaw cycles are not recommended. Multiwell cell culture plates should be prewarmed at 37°C for several hours before use for organoid culture. This will help the liquid BME‐organoid solution solidify on the culture plate.

### General Consumables and Equipment for Organoid Culture

General consumables include sterile 1.5‐, 15‐, and 50‐ml plastic tubes; multiwell plates in different well formats (6, 12, 24, and 48 wells); micropipette tips; and serological pipettes. General equipment includes a Class II biosafety cabinet, centrifuge, microcentrifuge, inverted light microscope, and 5% CO_2_ incubator at 37°C.

## Basic Protocol 1 ESTABLISHMENT OF HUMAN SMALL INTESTINE AND COLON ORGANOID CULTURES FROM FRESH BIOPSIES

This protocol describes a method for establishing human intestinal ASC‐derived organoid cultures from fresh biopsies of small intestine or colon. Tissue is dissociated into small epithelial fragments that are embedded into extracellular matrix “domes” using basement membrane extract (BME) and then supplemented with medium containing growth factors for organoid expansion.

Small intestine and colon biopsies are both suitable organoid culture sources. Because organoids maintain region‐specific characteristics, pooling biopsies from different intestinal regions or different donors is not recommended. In the case of CRC biopsies, a paired normal sample should be collected from surrounding healthy tissue.

### Materials

Fresh tissue biopsy from human small intestine or colon (healthy or tumor tissue)
70% (v/v) ethanol
AdvDMEM+++ (see recipe)
Primocin (InvivoGen, cat. no. ant‐pm‐1)
Collagenase type II (Sigma‐Aldrich, cat. no. C9407‐1)
Y‐27632 Rho kinase inhibitor (RhoKi; Abmole, cat. no. Y‐27632)
Cultrex reduced‐growth‐factor basement membrane extract (BME), type 2, Pathclear (R&D Systems/Bio‐Techne, cat. no. 3533‐001)
Red blood cell lysis solution (Gibco, cat. no. A1049201)
Expansion medium (see recipe)

15‐ and 50‐ml Falcon tubes
10‐cm Petri dish
Disposable scalpels (VWR, cat. no. HERE1110810)
Parafilm (Sigma Aldrich, cat. no. P7793‐1EA)
Orbital shaker
100‐µm cell strainer (Green Bioresearch, cat. no. 542000)
5‐ml pipet
24‐well cell culture plate (Greiner Bio‐One, cat. no. 662 102)

### Prepare cell suspension

1 Collect tissue sample from the tissue sample source site in a 50‐ml Falcon tube containing 25 ml AdvDMEM+++ and 100 μg/ml Primocin. Keep at 4°C until use.
  Use 30 mg tissue for optimal organoid line establishment. For increased efficiency, isolate tissue within 24 hr after surgery. Avoid freezing the tissue, as this will result in cell death.
2 Start up the biosafety cabinet. Disinfect the work area surface and pipets by spraying with 70% ethanol and allow to air dry.
3 Prepare fresh digestion medium for each tissue sample: Aliquot 5 ml AdvDMEM+++ into a 15‐ml plastic tube and add 5 mg/ml collagenase type II and 10 µM RhoKi. Mix by inverting and store on ice until use.
4 Transfer tissue to a 10‐cm Petri dish.
  Optionally, excise a tissue piece and place in a cryovial. Snap‐freeze and store in liquid nitrogen for additional DNA/RNA analysis and/or fix tissue for histology.
5 Using two scalpels, mince tissue into pieces of ∼1 mm^3^. Add the 5 ml digestion medium and mix by pipetting.
6 Transfer digestion medium with tissue to a 15‐ml plastic tube. Rinse the dish with the medium, returning the medium and all tissue pieces to the tube.
7 Close tube lid and seal with Parafilm. Place tube tilted at a low angle on an orbital shaker. Digest tissue for 30 to 45 min at 37°C and 140 rpm.
  Check the turbidity of the solution regularly. If the tissue is dissociated (typically after ∼30 min), the solution will look turbid without any large clumps.
8 Pipet the digested tissue onto a 100‐µm cell strainer on a 50‐ml plastic tube. Use a 5‐ml pipet to help pass the cell suspension through the filter.
  Large tissue pieces will be retained on the filter.
9 Rinse the strainer with 5 ml AdvDMEM+++ and transfer the solution (total 10 ml) to a fresh 15‐ml plastic tube.
10 Centrifuge 5 min at 450 × *g*, 4°C. Aspirate the supernatant.
  If red blood cells are present (observed as a dark red pellet), resuspend the pellet in 3 ml red blood cell lysis buffer and incubate for 5 min at room temperature. After incubation, add 5 ml AdvDMEM+++ to the cell solution, centrifuge 5 min at 450 × g, 4°C, and aspirate the supernatant.
11 Wash pellet three times with 10 ml AdvDMEM+++. Each time, spin 5 min at 450 × *g*, 4°C, and aspirate the supernatant.
12 Resuspend final pellet in undiluted BME.
  The volume of BME depends on the pellet size. Usually, a pellet from ∼30 mg primary tissue is plated in 300 µl BME. Cells plated at the right density have enhanced efficiency of organoid culture establishment. Plating too densely will result in increased cell death at the core of the dome.

### Plate cells and grow as organoids

**Figure 1:**
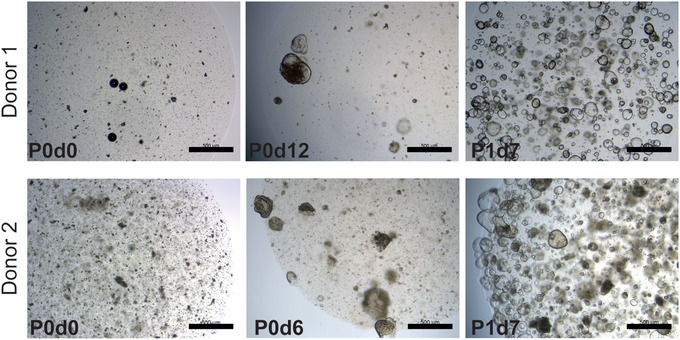
Establishment of human colon organoid lines from fresh biopsy of normal tissue. Representative images from two organoid lines. Cells are shown after establishment of cultures (left), before the first split (middle), and after the first split (right). P indicates passage number; d indicates days after seeding or passage; scale bars, 0.5 mm.

13 Plate 50 µl cell suspension per well as multiple ∼15‐µl droplets in a prewarmed 24‐well cell culture plate.
14 Place plate upside‐down in a 37°C, 5% CO_2_ cell culture incubator and allow droplets to solidify for 20 min.
  As the BME solidifies, the cells/organoids will spread over the volume of the BME, locating closer to the dome surface. This will maximize nutrient exchange and minimize organoid attachment to the plate. This also applies to Basic Protocol 2.
15 Carefully add 500 µl expansion medium supplemented with 10 µM RhoKi to each well and return the plate to the incubator.
  When establishing tumor lines, use expansion medium depleted of Wnt3A‐CM or Wnt surrogate. In this way, tumor cells with acquired Wnt pathway independence will be selected in culture.
16 After ∼1 week, expand organoids by mechanical disruption and splitting (see Basic Protocol 2).
  Due to donor‐to‐donor variability, the time required for a human intestinal organoid culture to reach confluence varies among organoid lines. Seven days is a standard estimation, but expansion time should be determined empirically for each individual line. Passaging organoids too early will result in increased cost in materials and suboptimal expansion speed. Passaging too late may result in spontaneously differentiating organoids, decreased cell viability, and suboptimal expansion. See Figure 1 for a reference for estimating the optimal time to perform the first split and expansion. This also applies to Basic Protocol 2.

## Basic Protocol 2 MECHANICAL SPLITTING, PASSAGE, AND EXPANSION OF HUMAN INTESTINAL ORGANOIDS

**Figure 2:**
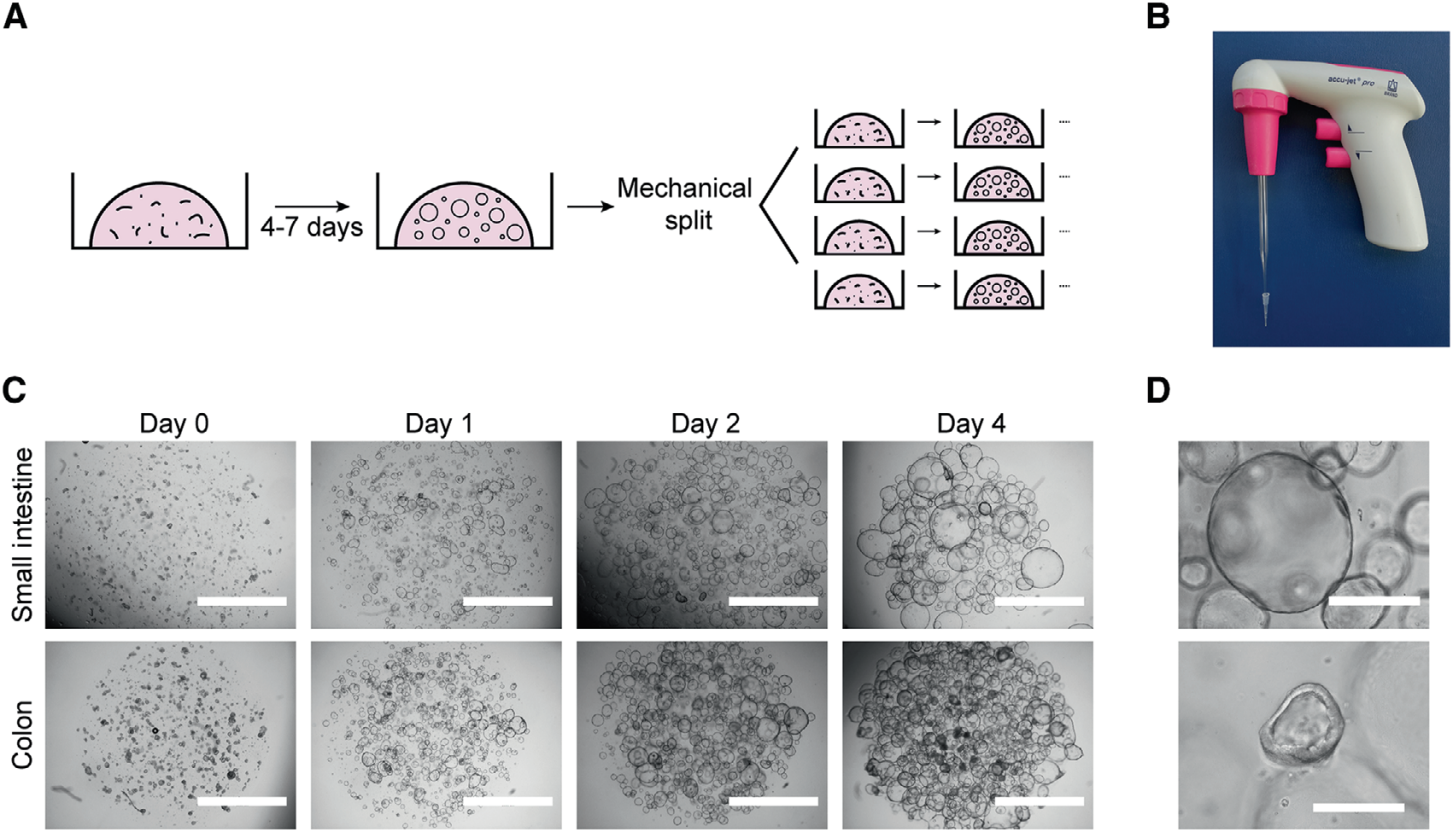
Mechanical splitting and expansion of human intestinal organoids. (**A**) Schematic of culture procedure. (**B**) Glass Pasteur pipette connected to a 10‐μl tip used for mechanical disruption of organoids. (**C**) Representative bright‐field microscopy images of organoid cultures at days 0, 1, 2, and 4 after mechanical split. Scale bars, 2 mm. (**D**) Examples of a healthy organoid (top), composed mostly of stem/transit amplifying cells, and a suboptimal organoid (bottom), with signs of differentiation (thickening of wall). Scale bars, 0.4 mm.

The process of mechanical splitting and passage is similar for organoids from human small intestine and colon due to the high similarity of these two organoid types. In short, organoids are recovered from the basement membrane matrix and broken mechanically into smaller fragments. The fragments are then resuspended in fresh BME and replated. They will self‐assemble as new organoids, and the stem cells and transit‐amplifying cells present in them will continue to proliferate, giving rise to a fully grown expanded organoid culture (Fig. 2A).

### Additional materials (also see Basic Protocol 1)

Established organoid culture (see Basic Protocol 1 if first passage, or this protocol if later passage)
Sterile plugged glass Pasteur pipette (VWR, cat. no. 14672‐400)
Sterile 10‐μl pipette tips
Mechanical pipetter

1 Remove medium from one well of a 12‐ or 24‐well plate of established organoid cultures.
  The process can be scaled up or down using different plate formats. For a guide to other formats, see Table 1.

**Table 1:** BME Volumes Used in Different Multiwell Plate Formats

Plate format	BME/well (μl)	Domes/well	Culture medium/well (ml)
6 wells	200	10‐15	2
12 wells	100	5‐7	1
24 wells	50	1‐3	0.5
48 wells	25	1	0.25
96 wells	5‐10	1	0.1

2 Using a P1000 pipette, flush the organoids with 1 ml ice‐cold AdvDMEM+++ to disrupt the BME droplets. Collect the organoids and transfer to a 15‐ml plastic tube containing 4 ml ice‐cold AdvDMEM+++.
3 Centrifuge the organoids 5 min at 450 × *g*, 4°C. Remove supernatant.
  During this step, undissolved BME often accumulates over the organoid pellet as a cloudy mesh. It is important to remove as much BME as possible without taking the organoids. If organoids do not separate from the BME, resuspend the pellet with the BME mesh in the AdvDMEM+++, incubate on ice for 10 min, and then repeat step 2.
4 Resuspend pellet in 1 ml ice‐cold AdvDMEM+++ using a P1000 pipette.
5 Attach a sterile 10‐μl pipette tip to the tip of a glass Pasteur pipette (Fig. 2B). Pass the organoids through the pipette opening five to ten times until large clumps are no longer visible.
6 Add 4 ml ice‐cold AdvDMEM+++ and centrifuge 5 min at 450 × *g*, 4°C. Remove supernatant.
  Foam formation at this step usually leads to loss of cells. To avoid this, pipette the cell solution against the middle part of the tube wall.
  Alternatively, mechanical disruption can be done using a fire‐polished glass pipette with the tip narrowed down to a diameter of ∼0.5‐1 mm using a Bunsen burner.
7 Resuspend pellet in 300‐400 μl BME using a P1000 pipette.
  Some residual AdvDMEM+++ is acceptable in the resuspension step, but the final BME concentration should be 75%‐100%.
  Avoid bubble formation, as this will render the BME droplet unstable and lead to its disruption before splitting time.
8 Dispense 100 μl suspension per well as multiple ∼15‐μl droplets in a prewarmed 12‐well culture plate (Fig. 2C).
  See Table 1 for dome numbers and volumes in different plate formats. The split ratio for a fully grown culture is typically 1:3 to 1:4 (of BME volume).
9 Flip the plate upside down and place plate in an incubator at 37°C and 5% CO_2_ for 20 min to let domes solidify.
10 Add 1 ml expansion medium and keep in the incubator, refreshing the medium every 2‐3 days.
  Under these conditions, organoids containing proliferating stem cells should look cystic and have a thin wall (Fig. 2D, upper panel). If organoids look dense and have a thick wall, they show signs of cell differentiation (Fig. 2D, lower panel), which will lead to loss of the cultures.
11 After 7 days, expand organoids further by repeating this process.
  As mentioned previously, the optimal time between passages varies and must be determined empirically for each line.

## DIFFERENTIATION OF HUMAN INTESTINAL ORGANOIDS

This protocol describes ways to differentiate organoids to the various cell types of the small intestinal and colonic epithelia. By withdrawing growth factors, which enforce a stem cell state, and adding components that skew differentiation trajectories, different types and proportions of mature cells can be obtained within 5 days. These mature cell types can be used for functional studies of the human gut epithelium.

### Additional materials (also see Basic Protocol 1)

Established human intestinal organoid culture (see Basic Protocol 2)
Differentiation medium (see recipe)

1 Aspirate medium from one well containing an established organoid culture grown to an average size of 100 µm (usually 5 days after splitting).
  Smaller organoids are sensitive to some of the small molecules used for differentiation at later steps.
2 Add 1 ml prewarmed AdvDMEM+++ and incubate at least 15 min in the 37°C incubator to allow diffusion of growth factors from the BME domes.
  For domes larger than 15 µl, the incubation time must be extended to allow proper diffusion.
  Volumes are based on a 12‐well plate. For other plate formats, see Table 1.
3 Aspirate medium from the well and add 1 ml prewarmed differentiation medium per well of a 12‐well plate. Return to incubator overnight.
  See Table 2 for different differentiation media and expected cell type composition.

**Table 2:** Intestinal Differentiation Media and Expected Cell‐Type Compositions*a*, *b*

Medium	Reference	Expected outcome	Special considerations
ENR	Sato et al. (2011)	Enterocytes, EECs, TACs, goblet cells	Generic differentiation medium for broad coverage of differentiated cell types, with particular enrichment of enterocytes
ENR+Notchi		Goblet cells	Goblet cell differentiation
ENR+MEKi+Notchi	Beumer et al. (2018)	EECs (crypt/lower villus state)	Enteroendocrine cell differentiation inducing a crypt hormone profile
ER+MEKi+Notchi+ BMP4+BMP2	Beumer et al. (2018)	EECs (upper villus state)	Enteroendocrine cell differentiation inducing an upper villus hormone profile

a For full composition of media, see Reagents and Solutions.

b Abbreviations: BMP, bone morphogenetic protein; EEC, enteroendocrine cell; ENR, EGF‐Noggin‐Rspo1; MEK, p38 MAP kinase; TAC, transit‐amplifying cell.

**Figure 3:**
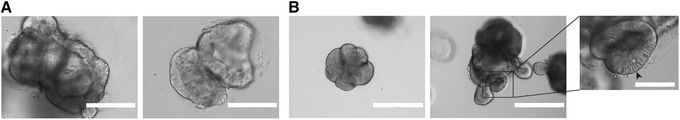
Differentiation of human intestinal organoids. (**A**) Representative images of organoids differentiated towards the enteroendocrine cell lineage as in Beumer et al. (2020). Scale bars, 0.2 mm. (**B**) Representative images of organoids differentiated towards the enterocyte lineage. Scale bars, 0.4 mm. Inset: Detail of differentiated organoid with enterocytes (elongated cells, arrowhead). Scale bar, 0.1 mm.

4 Refresh differentiation medium the next day to wash away any residual growth factors. Continue to refresh differentiation medium every 2‐3 days.
  As organoids differentiate, some cells undergo apoptosis and are shed to the organoid lumen. Together with more granular differentiated cells, this results in a darker appearance of the organoids, with thicker organoid walls and accumulation of debris in the organoid lumen. This can be used as an indicator of the progress of differentiation (Fig. 3A,B).
  After 5‐7 days, fully differentiated cells will be present in the organoids and the organoids can be used for downstream assays. Organoid viability can decrease substantially within days after terminal differentiation, so assays should be performed as early as possible for optimal results.

## Basic Protocol 3 CRYOPRESERVATION AND THAWING OF HUMAN ORGANOID CULTURES

This protocol details the steps for cryopreservation and thawing of intestinal and colon organoids. Cryopreservation is especially advisable when storing organoids for years in liquid nitrogen, but also for shipping organoid lines on dry ice. Briefly, organoids are freed from the BME and resuspended in freezing medium. To restart the cultures, organoids are carefully thawed, washed, and plated in BME domes. Thawed organoids are passaged according to Basic Protocol 2. After one passage, organoids should have normal growth characteristics and can be used for any downstream assay.

### Additional materials (also see Basic Protocol 1)

Established organoid culture (see Basic Protocol 2)
2× freezing medium (see recipe)
Cryopreservation tubes
Freezing container
37°C water bath

### Freeze organoids

1 Passage organoids 2‐3 days before cryopreservation (see Basic Protocol 2).
  For optimal recovery, small organoids in exponential growth phase should be frozen.
2 Remove medium from one well of a 12‐well plate of established organoid culture.
  The process can be scaled up or down using different plate formats. For a guide to other formats, see Table 1.
3 Using a P1000 pipette, flush the organoids with 1 ml ice‐cold AdvDMEM+++ to disrupt the BME droplets. Collect the organoids and transfer to a 15‐ml plastic tube containing 4 ml ice‐cold AdvDMEM+++.
4 Centrifuge organoids 5 min at 450 × *g*, 4°C. Remove supernatant.
  During this step, undissolved BME often accumulates over the organoid pellet as a cloudy mesh. It is important to remove as much BME as possible without taking the organoids. If this is not possible, resuspend the pellet in 10 ml ice‐cold AdvDMEM+++, incubate on ice for 10 min, and then repeat step 3.
5 Resuspend pellet in 1 volume of ice‐cold AdvDMEM+++ using a P1000 pipette, then add 1 volume of 2× freezing medium dropwise under constant shaking.
  The volume used in this step depends on the number of cryopreservation tubes being prepared and the organoid number per vial. For one densely plated well of a 12‐well plate, 500 µl each of AdvDMEM+++ and 2× freezing medium are recommended.
  Ensure slow addition of freezing medium to avoid an osmotic shock to the cells.
6 Transfer 1‐ml aliquots of organoid suspension to properly labeled cryopreservation tubes.
7 Place tubes in a freezing container and freeze at −80°C overnight.
8 Transfer tubes to a liquid nitrogen container.
  Organoids can be stored at −80°C for several weeks and in liquid nitrogen for years without major impairment of cell viability.

### Thaw stocks

9 Prepare AdvDMEM+++ and labeled 15‐ml tubes while organoids are still frozen to minimize thawing and processing times.
  Delays will lead to a decreased recovery rate.
10 Transport tubes of organoids from liquid nitrogen to a flow cabinet on dry ice.
11 Thaw tubes in a water bath at 37°C.
12 Transfer thawed organoid suspension to 15‐ml Falcon tubes (one per cryotube) and add 10 ml AdvDMEM+++ dropwise under constant shaking.
  Ensure slow addition of AdvDMEM+++ to avoid an osmotic shock to the cells.
13 Centrifuge 5 min at 450 × *g*, 4°C. Remove supernatant.
14 Resuspend each cell pellet in 100 μl BME using a P200 pipette, being careful to avoid bubble formation.
15 Dispense as ∼15‐µl drops in a well of a 12‐well cell culture suspension plate.
16 Place the plate upside‐down in the incubator for 20 min to let domes solidify.
17 Add expansion medium and keep in the incubator. Refresh medium every 2‐3 days.
  After 7 days, organoids can be further expanded as in Basic Protocol 2.

## Basic Protocol 4 IMMUNOFLUORESCENCE STAINING OF HUMAN INTESTINAL ORGANOIDS

This protocol describes a way to perform immunofluorescence staining compatible with fluorescence and confocal microscopy. For this purpose, organoids must first be released from the BME without disrupting their 3D architecture. Subsequent fixation, permeabilization, blocking, staining, and washes are performed with the organoids in suspension. This method can be applied to organoids that have been subjected to various experimental conditions.

### Materials

Established organoid culture (see Basic Protocol 2)
Fetal bovine serum (FBS, Sigma‐Aldrich, cat. no. F7524)
Cell Recovery Solution (Corning, cat. no. 354253)
Dispase (StemCell Technologies, cat. no. 07923; *optional*)
4% (v/v) formaldehyde in aqueous buffer
Permeabilization solution (see recipe)
Blocking solution (see recipe)
Mouse anti–human KI67 primary antibody (BD Pharmigen, cat. no. 550609)
PBS
Alexa Fluor 488 donkey anti‐mouse secondary antibody (Invitrogen, cat. no. A‐21202)
Phalloidin‐Atto 647N (Sigma‐Aldrich, cat. no. 65906)
DAPI
ProLong Gold Antifade Mounting solution (Thermo Fisher Scientific, cat. no. P36934)
Vaseline petroleum jelly
Clear nail polish

Sterile scissors
1000‐μl pipette tips
1.5‐ml microcentrifuge plastic tubes
Brightfield microscope
Tube roller
300‐μl low‐retention filter tips (Greiner Bio‐One, cat. no. 738 265)
96‐well black glass‐bottom plate (Greiner bio‐one, cat. no. 655892)
Microscope slides and cover slips

### Release organoids

1 Remove and discard medium from one well containing organoids.
  Starting with small organoids will help keep their structure intact. Starting with large and cystic organoids is more likely to result in disruption.
2 With sterile scissors, cut the opening of a 1000‐μl pipette tip 2‐3 mm from its end. Coat the tip and a 1.5‐ml microcentrifuge tube with FBS by pipetting 1 ml up and down once.
  Cutting the tip is required to avoid disrupting the organoid structure while pipetting. Coating with FBS minimizes adhesion of organoids to plastic surfaces.
3 Using the coated pipette tip, pipette 1 ml Cell Recovery Solution to the well, collect the organoids, and transfer to the coated microcentrifuge tube.
  Repeat steps 1‐3 for each well of cells to be stained (e.g., for different experimental conditions).
4 Incubate on ice for 20‐30 min, inverting regularly to prevent clumping and heterogeneous BME dissociation.
  Cell Recovery Solution dissolves the BME, freeing the organoids without disrupting their structure. Alternatively, the culture can be preincubated with dispase for 30 min at 37°C. Efficient dissolution of BME is key, as remaining BME can result in poor staining and higher background signal.
5 Monitor BME dissociation under a microscope, stopping when BME is sufficiently dissolved.
6 Let organoids settle by gravity to the bottom of the tube and remove the supernatant.

### Fix and permeabilize organoids

7 Add 1 ml of 4% formaldehyde and incubate 16 hr at 4°C (or 2 hr at room temperature) with constant rolling of the tube to ensure homogeneous fixation. Let organoids settle by gravity and remove supernatant.
  CAUTION: Formaldehyde is toxic. Avoid contact with skin and dispose of formaldehyde according to institutional and governmental safety rules.
8 Add 1 ml permeabilization solution and incubate with constant rolling for 30 min at 4°C. Let organoids settle by gravity and remove supernatant.
  Permeabilization time and temperature may need to be optimized for particular staining methods.

### Stain organoids

**Figure 4:**
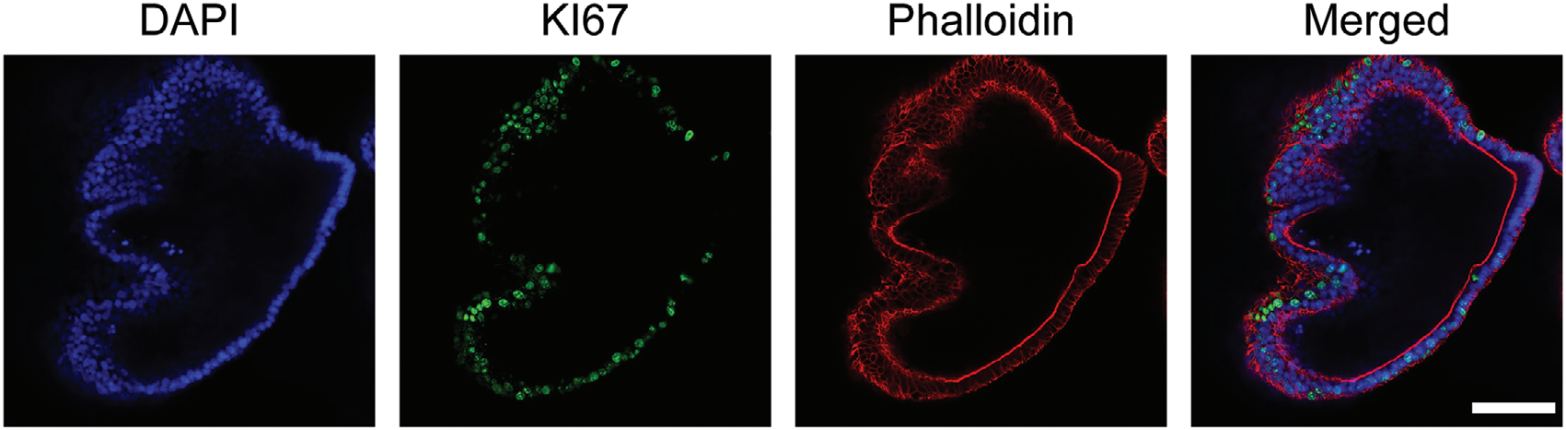
Confocal immunofluorescence images of human intestinal organoids cultured under expansion conditions. DAPI, nuclei; KI67, proliferation marker; phalloidin, F‐actin staining. Scale bar, 0.1 mm.

9 Add 1 ml blocking solution and incubate 15 min at room temperature under constant rolling. Let organoids settle by gravity and remove supernatant.
10 Add 200 µl of 500 ng/ml mouse anti–human KI67 primary antibody in blocking solution and incubate for 16 hr at 4°C under constant rolling. Let organoids settle by gravity and remove supernatant.
  If using a different primary antibody, the final concentration must be optimized.
11 Resuspend pellet in 1 ml PBS and incubate 10 min at room temperature with constant rolling. Let organoids settle by gravity and remove supernatant. Repeat wash two more times.
12 Add 200 µl of 4 µg/ml Alexa Fluor 488 donkey anti‐mouse secondary antibody, 10 nM phalloidin‐Atto 647N, and 2 µg/ml DAPI in blocking solution and incubate 1‐2 hr in the dark at room temperature with constant rolling.
  From this point, samples must be kept in the dark to protect the fluorophores from bleaching.
  If using a different secondary antibody, the final concentration must be optimized.
13 Wash three times as in step 11.
14 Wash once with Milli‐Q‐purified water to prevent crystal formation.
15 Cut the tip of a low‐retention 300‐μl tip 2‐3 mm from its end and then coat the tip with FBS by pipetting 200 μl up and down once.
  The FBS coating and low‐binding tips minimize the number of organoids lost due to adhesion to the plastic surface in the next step.
16a *For imaging in 96‐well plates*: Use the coated tip to resuspend organoids in 150‐200 μl PBS and transfer them to one well of a 96‐well black glass‐bottom plate. For optimal results, image samples immediately after staining (Fig. 4).

16b *For imaging on microscope slides*: Use the coated tip to resuspend organoids in 50 μl ProLong Gold mounting solution and transfer them to a microscope slide. Apply Vaseline at the edges of the slide so that the coverslip does not disrupt organoid structure after mounting. Seal the slide using nail polish.
  Slides can be stored at 4°C, maintaining the fluorescence signal for months.

## Basic Protocol 5 GENERATION OF SINGLE‐CELL CLONAL INTESTINAL ORGANOID CULTURES

**Figure 5:**
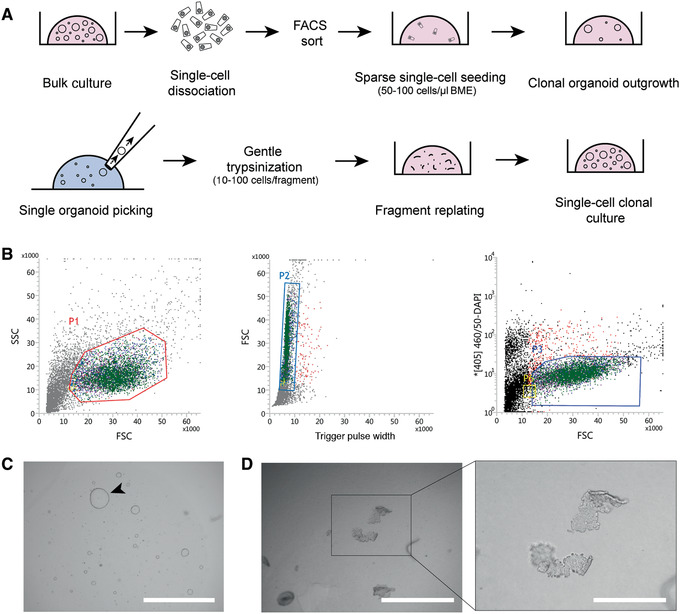
Clonal expansion of human intestinal organoid cultures. (**A**) Schematic of process. (**B**) Gating strategy for sorting of living cells used for clonal organoid outgrowth. Cells in P3 gate were sorted and seeded. (**C**) Single‐cell organoid outgrowth after 10 days. Arrowhead indicates organoid of proper size for picking. Scale bar, 2 mm. (**D**) Organoid fragments after trypsinization of a single picked organoid. Scale bars, 1 mm (left) and 0.4 mm (inset at right).

Many downstream applications of organoid cultures (e.g., CRISPR/Cas9 genetic engineering, whole‐genome sequencing) require generation of clonal cultures derived from single cells. To efficiently generate such cultures, organoids are first dissociated to single cells and seeded sparsely. After 10‐15 days, individual organoids are picked using a pipette tip, gently disrupted by enzymatic means, and reseeded. After this step, grown organoids can be mechanically split following Basic Protocol 2. For a general overview, see Figure 5A. This protocol can be carried out using established organoids from Basic Protocol 2 or organoids subjected to various experimental conditions, like genetic engineering, treatment with mutagens, or other experimental setups requiring WGS.

### Additional materials (also see Basic Protocols 1 and 4)

Established organoid culture (see Basic Protocol 2)
1× TrypLE Express Enzyme, phenol red (Gibco, cat. no. 12605036)

37°C water bath
Sterile plugged Pasteur glass pipettes and automated pipetter
Sterile 10‐μl pipette tips
Flow cytometry tubes with 35‐µm strainer cups (Corning, cat. no. 08‐771‐23)
Flow cytometer
12‐ and 48‐well cell culture plates (Greiner Bio‐One, cat. nos. 665 102 and 677 102)
Sterile 100‐mm cell culture dish

### Prepare organoids

1 Remove medium from one well of a 12‐well plate culture of established organoid culture.
  The process can be scaled up or down using different plate formats. For a guide to other formats, see Table 1.
2 Using a P1000 pipette, flush organoids from the well with 1 ml ice‐cold AdvDMEM+++ to disrupt the BME droplets. Collect the organoids and transfer to a 15‐ml centrifuge tube containing 4 ml ice‐cold AdvDMEM+++.
3 Centrifuge 5 min at 450 × *g*, 4°C. Remove supernatant.
  During this step, undissolved BME often accumulates over the organoid pellet as a cloudy mesh. It is important to remove as much BME as possible without taking the organoids. If this is not possible, resuspend the pellet in the AdvDMEM+++, incubate on ice for 10 min, and then repeat step 3.
4 Resuspend cell pellet in 1 ml prewarmed (37°C) TrpLE.
5 Incubate 5 min in a 37°C water bath, vortexing at regular intervals to increase dissociation. Check progress regularly under a microscope, and stop once the majority of fragments consist of single cells.
6 Coat a plugged glass Pasteur pipette with FBS to prevent cells from sticking to the glass. Connect a sterile 10‐μl tip to the Pasteur pipette and use to mechanically disrupt the organoid fragments to obtain single cells (also see Basic Protocol 2, step 5).
7 Check cell suspension under the microscope. If organoids are not dissociated into single cells, repeat steps 5 and 6.
  Long incubation times with TrpLE will lead to increased cell death. It is important to maximize the number of single cells while minimizing dead cells.
8 Add 4 ml ice‐cold AdvDMEM+++ with 10 µM RhoKi to the cells.
9 Move a small fraction of cells to another tube to use as a DAPI‐negative control in the FACS gating.
10 Centrifuge for 5 min at 450 × *g*, 4°C. Remove supernatant.
11 Resuspend cells in 0.5 ml ice‐cold AdvDMEM+++ supplemented with 10 µM RhoKi and 2 µg/ml DAPI. Omit DAPI in the negative control sample.
12 Pass cells through the strainer of a 35‐μm mesh‐cupped flow cytometry tube. Transport cells to the flow cytometer on ice and protected from light.
  DAPI will only stain apoptotic cells with disrupted cell membrane integrity.

### Perform sorting and start cultures from single live cells

13 Sort living cells (DAPI‐negative) into a 1.5‐ml microcentrifuge tube containing 0.5 ml AdvDMEM+++ supplemented with 10 µM RhoKi (Fig. 5B).
  - Use FSC and SSC to gate intact epithelial cells with intact morphology (Fig. 5B, left panel, P1).
  - On the gated population, use FSC and Trigger Pulse Width to select single cells and deplete cell duplets or triplets (Fig. 5B, middle panel, P2).
  - Use the [405]460/50‐DAPI channel to gate and sort living DAPI‐negative cells (Fig. 5B, right panel, P3).
  - At this step, note the number of cells sorted in the tube in order to calculate seeding cell density in the following steps.
  The cells not treated with DAPI can be used as a negative control in the FACS gating strategy (Fig. 5B).
  Sorting is important to make sure that single cells are used grow clonal cultures. It is very important if WGS will be performed, as loss of clonality prevents interpretation of WGS data.
  Alternatively, if cells have been subjected to CRISPR/Cas9 gene editing, cells can be passed through a 40‐µm cell strainer to increase the likelihood that dissociated single cells are seeded. After this, living cells are counted using a hemocytometer so that the seeding density can be estimated. While this approach is less stringent than sorting, it may enhance outgrowth efficiency and is acceptable if many clonal lines will be generated and screened later. Double‐picking strategies can be applied in order to increase the likelihood of obtaining clonal cultures.
14 Centrifuge cells 5 min at 450 × *g*, 4°C. Remove supernatant.
  The pellet may not be visible due to a low number of sorted cells. Be extremely careful when removing the supernatant, and do not touch the tube walls with the pipette tip.
  It is important that the BME droplets are solid after seeding to prevent dissociation before picking time and to avoid loss of organoid clones. To this end, make sure to remove as much supernatant as possible to increase the BME concentration.
15 Resuspend cells homogeneously in BME to a density of 50‐100 cells/µl. Pipette cells in ∼15‐µl domes in a 12‐well plate.
  The final cell concentration should be 50‐100 cells per µl BME in order to maximize organoid outgrowth and minimize organoid fusion afterwards. Sparse seeding will impair organoid outgrowth, while dense seeding could lead to organoid fusion and loss of clonality of the future culture.
16 Place the plate upside‐down in the incubator for 20 min to let the domes solidify.
17 Add expansion medium supplemented with 10 µM RhoKi and keep in the incubator, refreshing the medium (with RhoKi) every 2‐3 days.
  After 7‐14 days, single cells should have formed organoids (Fig. 5C). Due to donor‐to‐donor variability, some cultures will take longer. If this is the case, keep organoids expanding for an extra week before continuing with the next step.

### Isolate and expand clonal organoid cultures

18 Remove medium from the organoid well. Collect organoids in 1 ml Cell Recovery Solution and incubate on ice for 30 min.
  This should liberate organoids from the BME, making them easier to pick in the next step.
19 Pipette solution containing organoids in ∼500‐µl drops in a sterile 100‐mm cell culture dish.
  It is important that the drops not touch the dish walls, as organoids on the walls will not be visible under the microscope.
20 Place the plate under a brightfield microscope and open the lid.
  Avoid moving or speaking above the opened plate. Make sure to add Primocin to the expansion medium at step 26.
21 Using a P20 pipette, pick individual organoids from the drop and transfer each one to a separate 1.5‐ml microcentrifuge tube containing 300 µl TrypLE at 4°C.
  It is recommended to pick a high number of clonal organoids, because the outgrowth efficiency is not 100%. We recommend picking ∼20 organoids per condition in an optimal growth state.
22 Incubate in a 37°C water bath for 1 min.
23 Add 700 µl AdvDMEM+++ containing 10 µM RhoKi and pipette up and down three to five times using a P1000 pipette.
24 Centrifuge 5 min at 500 × *g*, 4°C. Remove supernatant.
  Again, the pellet may not be visible due to the low number of cells in the organoid. Be extremely careful when removing the supernatant, and do not touch the tube walls with the pipette tip.
25 Resuspend in 20 µl BME and seed in a well of a 48‐well plate (Fig. 4D).
26 Add expansion medium supplemented with 100 μg/ml Primocin and 10 µM RhoKi.
27 After organoids grow out, pass them as in Basic Protocol 2.

## Support Protocol 1 PRODUCTION OF Wnt3A CONDITIONED MEDIUM

Wnt3A‐CM is an important component of the human intestinal organoid expansion medium. Its role in activating the Wnt pathway is key to maintenance of the stem cell population. Thus, the production of high‐quality Wnt3A‐CM is essential for successful establishment and culture of human intestinal organoids.

### Materials

L‐Wnt‐3A cells from frozen cryovial (from a T25 flask cultured to 95%‐100% confluence) (provided upon request and after material transfer agreement)
DMEM++ (see recipe)
Zeocin (Gibco, cat. no. R25001)
1× TrypLE Express Enzyme, phenol red (Gibco, cat. no. 12605036)
HEK 293‐STF cells (ATCC, CRL‐3249)

T175 cell culture flasks (Greiner Bio‐One, cat. no. 661160)
37°C, 5% CO_2_ cell culture incubator
145‐mm cell culture dishes (Greiner Bio‐One, cat. no. 639160)
0.22‐µm Stericup‐GP filter (polyethersulfone, 500 ml, radio‐sterilized; Millipore, cat. no. SCGPU05RE)

1 Start L‐Wnt‐3A cell culture from a frozen cryovial. Seed cells in a T175 flask with 20 ml DMEM++ supplemented with 125 µg/ml Zeocin. Grow at 37°C and 5% CO_2_ until the culture reaches 90%‐100% confluence (~3‐4 days).
  A key component of DMEM++ medium is the FBS, to which L cells are very sensitive. Different batches of FBS should be tested to find one that efficiently supports growth of both L cells and organoids.
2 Split cells to two T175 flasks using TrypLE and grow cells in DMEM++ with 125 µg/ml Zeocin at 37°C and 5% CO_2_ until they reach 90%‐100% confluence (~3‐4 days).
3 Split cells to a total of twelve T175 flasks. Add DMEM++ supplemented with 125 µg/ml Zeocin to two flasks and use these for a new round of Wnt3A‐CM production (step 2). Add DMEM++ without Zeocin to the other ten flasks and use these for steps 4‐7.
  L‐Wnt‐3A cells should only be used until passage 10‐12.
4 Allow cultures in the ten flasks to grow to confluence (~1 week).
5 Trypsinize cultures, pool the cells, and seed in sixty 145‐cm^2^ dishes in 20 ml DMEM++ without Zeocin per plate. Incubate cells for 1 week at 37°C and 5% CO_2_.
6 Harvest medium and centrifuge for 5 min at 450 × *g*, 4°C.
7 Collect supernatant and pass through a 0.22‐µm Stericup‐GP filter.
  It is important to remove all L‐Wnt‐3A cells from the conditioned medium, as they will otherwise overgrow the organoids when the Wnt3A‐CM is used for organoid culture.
8 Test the quality of every batch of Wnt3A‐CM using a luciferase‐based assay in the HEK 293T‐STF Wnt reporter cell line (Xu et al., 2004).
  As a reference, an optimal batch induces 15‐ and 300‐fold increases in signal when used at concentrations of 12.5% and 50% (v/v), respectively. Using Wnt3A‐CM at 1%‐2% (v/v) should give a signal similar to background.
  Freshly prepared Wnt3A‐CM can be stored at 4°C for long periods of time (>6 months).

## Support Protocol 2 PRODUCTION OF Rspo1 CONDITIONED MEDIUM

Rspo1‐CM is used to prepare organoid medium as well as all of the differentiation media described here. The presence of Rspo1 in organoid expansion medium results in a synergistic increase in Wnt pathway activation levels compared to those achieved by Wnt3A‐CM alone. Thus, Rspo1‐CM is another key component of the medium used for establishment and expansion of human intestinal organoids.

### Additional materials (also see Support Protocol 1)

293T‐HA‐RspoI Fc cells from frozen cryovial (from a T25 flask cultured to 95%‐100% confluence) (R&D Systems, cat. no. 3710‐001‐01)
T75 cell culture flasks (Greiner Bio‐one, cat. no. 658170)

1 Start 293T‐HA‐RspoI Fc cell culture from a frozen cryovial. Seed cells in a T75 flask with 20 ml DMEM++ supplemented with 300 µg/ml Zeocin. Grow at 37°C and 5% CO_2_ until the culture reaches 90%‐100% confluence.
2 Split cells to two T175 flasks using TrypLE and grow cells in DMEM++ with 300 µg/ml Zeocin at 37°C and 5% CO_2_ until they reach 90%‐100% confluence.
3 Split cells to a total of twelve T175 flasks. Add DMEM++ supplemented with 300 µg/ml Zeocin to two flasks and use these for a new round of Rspo1‐CM production (step 2). Add DMEM++ without Zeocin to the other ten flasks and use these for steps 4‐6.
  293T‐HA‐RspoI Fc cells should only be used until passage 10‐12.
4 Grow cells in the ten flasks to 75% confluence (2‐3 days).
5 Remove DMEM++ from the culture and add 50 ml serum‐free AdvDMEM+++ without Zeocin per flask. Incubate cells for 1 week at 37°C and 5% CO_2_.
  During the week, the culture medium will acquire a yellow color. The medium should not be refreshed, because Rspo1 is being secreted during this time.
6 Harvest medium and centrifuge for 5 min at 450 × *g*, 4°C.
7 Collect supernatant and pass through a 0.22‐µm Stericup‐GP filter.
  It is important to remove all 293T cells from the conditioned medium, as they will otherwise overgrow the organoids when the Rspo1‐CM is used for organoid culture.
8 Test the quality of every batch of Rspo1‐CM using a luciferase‐based assay in the HEK 293T‐STF Wnt reporter cell line.
  As a reference, an optimal batch induces 100‐ and 600‐fold increases in signal over background levels when used at concentrations of 0.25% and 2.5% (v/v), respectively, when used in combination with 12.5% (v/v) Wnt3A‐CM. In the absence of Wnt3A‐CM, Rspo1‐CM does not significantly activate the Wnt pathway. However, its presence there contributes to increased viability of terminally differentiated organoids.
  The process can be scaled up if larger volumes of Rspo1‐CM are required. The ratio of medium volume to culture surface should be maintained.
  Freshly prepared Rspo1‐CM can be stored at −20°C for 6 months. The CM should be dispensed in aliquots adequate to your needs before freezing. Once thawed, store at 4°C and use preferably within 4 weeks. Avoid repeated freeze‐thaw cycles.

## Support Protocol 3 EXTRACTION OF RNA FROM INTESTINAL ORGANOID CULTURES

This protocol can be used with established organoid cultures, including those subjected to various experimental conditions.

### Materials

Established organoid culture (see Basic Protocol 2)
Column‐based extraction kit (RNAeasy Mini Kit, Qiagen, cat. no. 74104)
Freshly prepared 70% (v/v) ethanol
RNase‐free DNase set (Qiagen, cat. no. 79254)
RNase‐free 1.5‐ml microcentrifuge tubes

1 Remove medium from organoid well and dissolve the BME drop containing the organoids directly in 350 µl RLT buffer from the RNAeasy Mini Kit. Pipet several times up and down and transfer the suspension to a 15‐ml plastic tube.
  The starting material should be at least one full well of a 24‐well culture plate. To ensure there is enough RNA material, it is preferable to use one full well of a 12‐well plate. If organoids are cultured under differentiation conditions, expect the yield to decrease and use increased amounts of organoids for RNA assessments.
2 Mix the resulting solution 1:1 (v/v) with freshly prepared 70% ethanol and vortex.
3 Isolate RNA according to the manufacturer's instructions, starting with step 4 of “Protocol: Purification of Total RNA from Animal Cells Using Spin Technology” from the *Qiagen RNAeasy Handbook Guide* (https://www.qiagen.com/ie/resources/resourcedetail?id=14e7cf6e‐521a‐4cf7‐8cbc‐bf9f6fa33e24&lang=en).
  Perform optional DNase digestion after step 5 of the protocol, as indicated in Appendix D (“Optional On‐Column DNase Digestion with the RNase‐Free DNase Set”) of the guide.
4 Elute in 30 µl RNase‐free water.
  Elution volume depends on the amount of starting material and should be adjusted accordingly.

## REAGENTS AND SOLUTIONS

### AdvDMEM+++

Advanced Dulbecco's Modified Eagle Medium/F12 (Gibco, cat. no. 12634‐010)
10 mM HEPES (Gibco, cat. no. 15630‐056)
1× GlutaMAX Supplement (Gibco, cat. no. 35050‐038)
100 U/ml penicillin‐streptomycin (Gibco, cat. no. 15140163)
Store up to 1 month at 4°C

### Blocking solution

Phosphate‐buffered saline (PBS)
0.1% (v/v) Tween‐20
2% (v/v) donkey serum
Prepare fresh before use

### Differentiation medium

*For base ENR medium*:

AdvDMEM+++ (see recipe)
1× B‐27 Supplement (50×, serum free, Life Technologies, 17504‐044)
20% (v/v) Rspo1‐CM (see Support Protocol 2)
2% (v/v) Noggin conditioned medium (U‐Protein Express, N002)
1.25 mM *N*‐acetylcysteine (Sigma‐Aldrich, A9165)
50 ng/ml epidermal growth factor (EGF, Peprotech, AF‐100‐15)
Store up to 1 month at 4°C

*Before use, add differentiation components*:

10 μM Notch inhibitor DAPT (Sigma‐Aldrich, D5942)
0.1‐1 μM MEK inhibitor PD0325901 (Sigma‐Aldrich, PZ0162)
50 ng/ml BMP2 (Peprotech, 120‐02C)
50 ng/ml BMP4 (Peprotech, 120‐05ET)
  See Table 1 for selection of appropriate medium. For ENR+Notchi, add DAPT to base medium. For ENR+MEKi+Notchi, add PD0325901 and DAPT to base medium. For ER+MEKi+Notchi+BMP2+BMP4, add all four components and omit Noggin CM. Additional differentiation components should be added fresh to the medium prior use.

### DMEM++

1× DMEM + GlutaMAX‐I (Gibco, cat. no. 31966‐021)
100 U/ml penicillin‐streptomycin (Gibco, cat. no. 15140163)
10% (v/v) fetal bovine serum (FBS; Sigma‐Aldrich, cat. no. F7524)
Store up to 1 month at 4°C

### Expansion medium

AdvDMEM+++ (see recipe)
1× B‐27 Supplement (50×, serum free, Life Technologies, 17504‐044)
50% (v/v) Wnt3A‐CM (see Support Protocol 1) *or* 0.5 nM Wnt surrogate (U‐Protein Express, N001)
20% (v/v) Rspo1‐CM (see Support Protocol 2)
2% (v/v) Noggin conditioned medium (U‐Protein Express, N002)
1.25 mM *N*‐acetylcysteine (Sigma‐Aldrich, A9165)
10 mM nicotinamide (Sigma‐Aldrich, N0636)
10 μM p38 inhibitor SB202190 (Sigma‐Aldrich, S7076)
50 ng/ml epidermal growth factor (EGF, Peprotech, AF‐100‐15)
0.5 μM ALK5 inhibitor A83‐01 (Tocris/Bio‐Techne, 2939)
1 μM prostaglandin E2 (PGE2, Tocris/Bio‐Techne, 2296)
Store up to 1 month at 4°C
  See Strategic Planning and Critical Parameters for discussions on the use of Wnt ligands. Omit Wnt ligands when culturing CRC‐derived organoids.

### Freezing medium, 2×

10 ml DMSO (Sigma‐Aldrich, cat. no. D2650)
40 ml FBS (Sigma‐Aldrich, cat. no. F7524)
Store up to 1 month at 4°C

### Permeabilization solution

Phosphate‐buffered saline (PBS)
0.5% (v/v) Triton X‐100
2% (v/v) donkey serum (Bio‐Rad, C06SB)
Prepare fresh before use

## COMMENTARY

### Background Information

The initial development of ASC‐derived organoids occurred hand‐in‐hand with knowledge obtained on the signaling pathways governing development of the adult intestinal epithelium. The intestinal crypt‐villus (small intestine) or crypt (colon) structural units are hallmarks of adult stem cell functioning. Intestinal stem cells—marked by expression of the G‐protein coupled receptor Lgr5—reside at the bottom of the crypts and are able to replenish the entire intestinal epithelium within a short turnover time of ∼5 days. Lgr5^+^ cells that reside at the bottom of the crypts actively divide, generating transit‐amplifying cells. These cells proliferate and give rise to all functional epithelial cell types that carry out the essential intestinal functions (e.g., enterocytes, Paneth cells, enteroendocrine cells, goblet cells, tuft cells, and—at the Peyer's patches—M cells). The population dynamics of these cells are tightly governed by the action of four signaling pathways, namely Wnt, epidermal growth factor (EGF), Notch, and bone morphogenetic protein (BMP) (Clevers, 2013).

ASC‐derived intestinal organoids rely on the infinite ability of epithelial Lgr5^+^ stem cells to divide and repopulate the entire intestinal epithelium. The initial development of the technology was guided by knowledge on the niche factors that promote the stem cell state at the bottom of the crypt (Sato et al., 2009, 2011). By providing Wnt and EGF agonists and BMP inhibitors in the medium, these stem cells can be maintained and expanded in culture indefinitely. In the case of mouse small intestinal organoids, Paneth cells are a source of Wnt ligands that allow organoids to be cultured in the absence of an exogenous Wnt source. This inherently creates a local Wnt gradient around Paneth cells, giving rise to the characteristic budding structures of mouse small intestinal organoids. For human intestinal organoids, ectopic Wnt ligands need to be added to the medium, as Paneth cells do not produce as much Wnt as in the mouse. In turn, this leads to the characteristic cystic structures of human small intestine and colon organoids.

### Critical Parameters and Troubleshooting

It is essential to establish organoid cultures from fresh biopsies after surgery and to avoid freezing the tissue. A substantial delay after surgery and freezing of the tissue can both result in failure to obtain organoids. Additionally, the quality (e.g., low proportion of epithelial cells, high proportion of necrotic cells) of the tissue biopsies influences the efficiency of organoid outgrowth.

One of the most critical parameters regarding the culture of human intestinal organoids is the source of Wnt pathway agonists and the level of Wnt pathway activation that they achieve. High levels of Wnt activation will result in cystic organoids that maintain most of their cells in a stem cell or transit‐amplifying state, allowing indefinite expansion of the culture. On the other hand, if a suboptimal Wnt agonist source is used, stem cells will start to differentiate, leading to loss of the organoid culture after some passages. Wnt3A‐CM is the most established source of Wnt agonist, and a protocol for its production is included here. However, batch‐to‐batch variability in Wnt activation activity mandates regular quality controls of this reagent. In order to overcome this problem, it is recommended to use a combination of different Wnt3A‐CM batches, and to perform Wnt activity assays using a reporter cell line (ATCC, CRL‐3249). Additionally, the recent development of synthetic Wnt agonists (Janda et al., 2017; Miao et al., 2020) has improved and facilitated the culture of human intestinal organoids, particularly when growing human colon organoids from single cells.

When using organoids for experiments, it is crucial to consider the cell type composition. Organoids cultured in expansion medium will predominantly consist of stem and transit‐amplifying cells. These are useful in stem cell and cancer studies, but many experiments on healthy gut physiology require mature cell types. These can be obtained within 5 days using tailored differentiation media for all major mature cell types. It is crucial to validate differentiation success by quantitative real‐time PCR for cell‐type marker genes or antibody‐based staining. Some of the most widely used markers for different intestinal epithelial cell types are: AXIN2 or OLFM4 for stem cells, FABP1 or Villin for enterocytes, CHGA for enteroendocrine cells, LYZ for Paneth cells, and MUC2 for goblet cells. Beyond the medium recipes provided here, other differentiation strategies can be applied (Beumer et al., 2020; Fujii et al., 2018) but are beyond the scope of this protocol.

Another matter of high importance is the effect of cell density on the ability to expand the organoid culture. The right seeding density (1) enables paracrine signaling support for organoid growth, (2) leaves space for organoids to grow, (3) avoids excessive consumption of media components, and (4) ensures adequate diffusion of growth factors to the core of BME domes. The seeding density has an especially crucial effect in the single‐cell clonal expansion step. Seeding that is too sparse may greatly reduce outgrowth efficiency, whereas a high seeding density increases the risk of organoid fusion and thereby loss of clonality. This is especially relevant in experiments involving whole‐genome sequencing, where the cost per genome is high. It is recommended to maintain organoids seeded at the density indicated in Basic Protocol 5 (Fig. 5). As mentioned previously, there is considerable inter‐donor variability in organoid line density preference and tolerance. Some lines display expansion potential even at suboptimal density, whereas others tend to differentiate when plated too sparsely and lose viability when plated too densely.

Another important factor to consider is the addition of Rho kinase inhibitor in all steps at which organoids are dissociated to single cells, both during passage and once seeded. This will block anoikis, which otherwise occurs due to the loss of cell‐cell contact.

Using low‐attachment pipette tips and coating with FBS prior to pipetting prevents organoids from attaching to the pipette tip and thus subsequent material loss. This is particularly important when organoids are transferred in the first and last steps of whole‐mount immunofluorescence staining (Basic Protocol 4), but can also be advisable when establishing organoid lines from limited material.

For a troubleshooting guide, see Table 3.

**Table 3:** Troubleshooting Guide

Observation	Possible cause(s)	Suggested solutions
Organoids do not grow after isolation and establishment of line	Low quality; biopsy was incubated too long at 4°C or was frozen	Minimize time between biopsy and establishment of line
	Biopsy was incubated too long in dissociation medium	Try to minimize the dissociation time
	Did not add RhoKi to the expansion medium	Add RhoKi to medium
Organoids stop growing	Wnt3A‐CM and Rspo1‐CM batches do not provide optimal Wnt pathway activation	Test batches in organoids cultures before using in relevant cultures
		Alternatively, test media using Wnt reporter cell lines
Organoid biomass lost at passage	Organoids trapped in foam generated during mechanical dissociation or in the filter of the Pasteur pipette	Avoid foam formation by pipetting against tube wall; be careful not to let suspension reach the pipette filter when performing mechanical disruption
Organoids lost during staining	Organoids did not settle by gravity	Make sure that most of the organoids have settled before removing supernatant; a quick spin in a benchtop centrifuge (2 s) may help
	Organoids adhered to plastic tip during last transfer	Use low‐binding tips coated with FBS
Organoids fail to differentiate (maintain cystic morphology)	Stem cell factors were not washed away properly before differentiation	Starting with a fresh organoid culture in expansion medium, increase the number of 15‐min wash cycles (up to three). Make sure to change medium 1 day after induction of differentiation.
	Differentiation medium was not freshly prepared	Make fresh medium prior to experiment
	Differentiation components are missing	Check that all components were added to differentiation medium
Excessively dark organoid morphology after differentiation	Cell death due to growth pathway inhibition/lack of expansion signals	Adjust inhibitor concentrations; assess other organoid lines for better differentiation capacity
Background after antibody staining	Residual BME attached to organoids after collection	Incubate longer in Cell Recovery Solution; use dispase as an alternative method
	Antibody concentration not optimal	Optimize antibody concentration
	Permeabilization not optimal	Adjust permeabilization time
Low single‐cell outgrowth efficiency	Not enough Wnt activation	Optimize Wnt source and concentration
	Seeding density too low	Optimize seeding density
	Trypsinization time was too long	Stop trypsinization after a maximum of 5 min
Non‐clonal organoid outgrowth (detected by WGS or imaging)	Single cells seeded at too high density, leading to organoid fusion	Seed cells at lower density and check daily under a microscope; make sure to pick only one organoid at a time

### Time Considerations

The amount of hands‐on time dedicated to these techniques is not extensive, although organoid work is more dedicated and requires more attention than work with cell lines. Additionally, organoids grow considerably more slowly than standard cell lines. The time required to perform all these techniques depends on the number of lines being processed in parallel.

Typically, establishing one organoid line from a biopsy takes ∼3 hr, with 1.5 hr hands‐on time. It normally takes an additional 2‐4 weeks and two passages before enough biomass is generated for cryopreservation and experiments. Performing mechanical passage of a single organoid line could take ∼1 hr, with 15 min hands‐on time. In general, the time required for organoids to expand after passage is 1 week, although donor‐to‐donor variability should be taken into consideration. Differentiation of organoids takes ∼30 min of hands‐on time and 5 days of incubation. The staining protocol for organoid immunofluorescence usually takes 2 days if fixation is performed overnight. If fixation is performed for 2 hr at room temperature, it can be completed within 1 day. Establishing single‐cell clonal organoid lines is a long process. In some cases, it can take 2 months before a fully clonal culture is established (one full well of a 12‐well plate), or even longer depending on the donor. The hands‐on time required for the initial step of single‐cell culture establishment is ∼2 hr. Single‐cell outgrowth to organoids can take 2 or 3 weeks depending on the organoid line. The time required to pick organoids depends highly on the number of clones to be picked. Picking and passing 24 clones from a single condition usually takes 30‐60 min. Expansion of these clones in 48‐well plates requires 1‐2 weeks, after which another passage (1‐2 weeks) will be required to obtain one full well of a 12‐well plate per clonal culture. RNA extraction from organoids takes 30‐60 min. DNA extraction from organoids takes ∼3 hr total time. The production of Wnt3A and Rspo1 conditioned medium takes 12 days for one batch. If more batches are produced in parallel, production time will increase to ∼20 days.

### Conflicts of Interest

H.C. is the inventor on several patents related to organoid technology; his full disclosure is given at https://www.uu.nl/staff/JCClevers/.

### Author Contributions

**Cayetano Pleguezuelos‐Manzano**: Methodology; writing‐original draft; writing‐review & editing. **Jens Puschhof**: Methodology; writing‐original draft; writing‐review & editing. **Stieneke van den Brink**: Methodology. **Veerle Geurts**: Methodology. **Joep Beumer**: Methodology. **Hans Clevers**: Funding acquisition; project administration; supervision.
